# Supplementary material for: Splice-Junction-Based Mapping of Alternative Isoforms in the Human Proteome
Source: Cell Rep. Author manuscript; Available in PMC 2020 Jan 15. (PMC6961840; doi:10.1016/j.celrep.2019.11.026)
Supplement: 2 [file NIHMS1546469-supplement-2.zip › DF1/PXD010154/Heart/Heart_90_CDK16_WTPSSKPQTEAPAGSGWR.pdf]

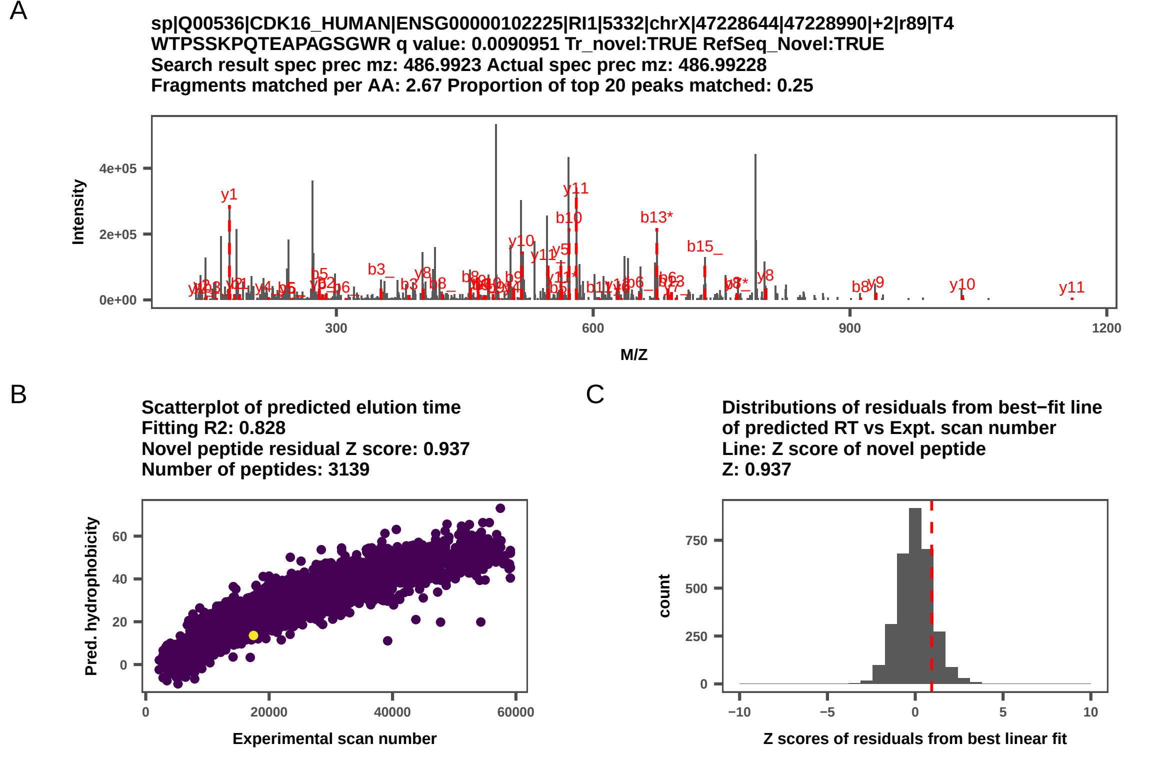
A
sp|Q00536|CDK16_HUMAN|ENSG00000102225|RI1|5332|chrX|47228644|47228990|+2|r89|T4
WTPSSKPQTEAPAGSGWR q value: 0.0090951 Tr_novel:TRUE RefSeq_Novel:TRUE
Search result spec prec mz: 486.9923 Actual spec prec mz: 486.99228
Fragments matched per AA: 2.67 Proportion of top 20 peaks matched: 0.25
Intensity
4e+05
2e+05
0e+00
y1
y11
b10
b13*
y10
b15_
y8
y9
y10
y11
300
600
900
1200
M/Z
B
Scatterplot of predicted elution time
Fitting R2: 0.828
Novel peptide residual Z score: 0.937
Number of peptides: 3139
Pred. hydrophobicity
60
40
20
0
0
20000
40000
60000
Experimental scan number
C
Distributions of residuals from best−fit line
of predicted RT vs Expt. scan number
Line: Z score of novel peptide
Z: 0.937
count
750
500
250
0
−10
−5
0
5
10
Z scores of residuals from best linear fit
